# Private sector engagement strategies with implications for NCD prevention and control: focus on ten international organisations

**DOI:** 10.1186/s41256-025-00448-4

**Published:** 2025-09-27

**Authors:** Téa E. Collins, Svetlana Akselrod, Daria Berlina, Amanda Karapici, Flaminia Ortenzi, Fatima Bashir, Luke N. Allen

**Affiliations:** 1 Global NCD Platform, World Health Organization, Geneva, Switzerland; 2 Blavatnik School of Government, University of Oxford, Oxford, UK; 3 Global Primary Care and Future Health Systems, University of Oxford, Oxford, UK

**Keywords:** Private sector engagement, Private sector, Noncommunicable diseases, Global health, Governance

## Abstract

**Background:**

International organisations and development agencies have important roles to play in addressing noncommunicable diseases (NCDs) and their risk factors at the nexus of health, socioeconomic, and environmental development challenges. Much of this work occurs through direct engagement with the private sector. We aimed to assess the types of private sector engagement (PSE) approaches and the degree of alignment across ten major international organisations whose work is critical to achieving global NCD and mental health goals.

**Methods:**

We examined the publicly available PSE strategy documents for a purposive sample of ten major international development partners. We obtained copies of each organisation’s publicly available PSE policy documents and extracted data on the stated purpose, processes, and types of engagement. We used thematic analysis and triangulation to identify areas of agreement, dissonance and silence across the policy approaches.

**Results:**

Whilst all PSE documents emphasised the importance of conducting due diligence, they varied widely in their approach to the risk of engagement and the sophistication of potential conflict of interest management strategies. Many documents were silent on prohibited industries, managing reputational risks, and guidance to Member States. The proactive engagement stance in USAID and World Bank policy documents contrasted starkly with more conservative approaches advanced by UNDP, FAO, and WHO.

**Conclusions:**

The core practices of conducting due diligence and risk mitigation are common to all of the major international organizations we assessed, however, the framing, content, and PSE processes vary widely. The potential impact of these findings is that WHO and other partners can focus on adopting common approaches wherever possible for greater coherence and smoother coordination across the wider development system.

## Introduction

Noncommunicable diseases (NCDs) cause an estimated 41 million deaths each year, and mental health conditions account for 16% of global disability-adjusted life years (DALYs) at an economic cost of approximately USD 5 trillion [1]. With the adoption of the 2030 Agenda for Sustainable Development, NCDs were recognized as a development priority interlinked with many other sustainable development goals (SDGs)—requiring a multisectoral and multi-stakeholder response, as well as greater policy coherence across all government sectors and the United Nations (UN) system, and other international organisations [2–4]. NCDs share important risk factors with other pressing global development challenges impacting health, including poverty, climate change and inequalities [5, 6]. Unhealthy diet, physical inactivity, and the use of tobacco and alcohol predispose to a range of infectious and non-infectious diseases [7]. NCDs are a major source of catastrophic health expenditure, and these conditions commonly co-exist with other health problems [8]. The NCD agenda also overlaps with those of HIV, tuberculosis, malaria, and broader development concerns, and many international organisations that contribute significantly to the global NCD agenda operate under non-NCD mandates [9].

The private sector is centrally important to achieving global NCD and mental health goals [10, 11]. The 2030 Agenda recognises the important role of businesses in shaping sustainable development and encourages all private sector entities to contribute their expertise, creativity, and resources to the SDGs as part of a broad call to “unlock the transformative power of the private sector” [2, 12]. The need for multistakeholder collaboration is highlighted by SDG17: “strengthen the means of implementation and revitalise the global partnership for sustainable development.” In particular, Target 17.16 calls for enhancing multi-stakeholder partnerships to mobilise knowledge, expertise, technology and financial resources towards the achievement of the SDGs and Target 17.17 specifically focuses on promoting effective public, public–private, and civil society partnerships [2, 12].

Collins et al. have argued that whilst private sector engagement has a “fraught track record,” it remains underutilised in the fight against NCDs, and note that a blanket response is not appropriate given the heterogeneity of the sector [13]. Given that much of the existing private sector engagement (PSE) to address NCDs occurs through international organisations and development agencies, it’s important that these agencies have robust and well-aligned PSE approaches, as well as consistent guidance offered to their Member States and partners. A recent UN Secretary-General report has also emphasised the importance of such robust and coordinated approaches for effective engagement with the private sector [14], in line with SDG 17 to “enhance policy coherence for sustainable development” [12].

In this article, we aimed to assess PSE alignment across major international health and development organisations whose work is critical for achieving the NCD and mental health targets of the 2030 Agenda. Our objectives were to analyse the content and alignment of each organisation’s PSE policy documents using qualitative document review.

## Methods

### Setting and research design

This study was built on a previous internal mapping exercise conducted by the World Health Organization Global NCD Platform and the Centre for Strategic and International Studies (CSIS) to identify the major international organisations and development agencies whose work impacts the attainment of the NCD and mental health-related Sustainable Development Goals: the Food and Agriculture Organisation (FAO), Gavi the Vaccine Alliance (Gavi), the Global Fund, the Swiss Development Agency (SDC), the UN Refugee Agency (UNHCR), the UN Development Programme (UNDP), the UN Children’s Fund (UNICEF), the US Agency for International Development (USAID), the World Bank, and the World Health Organisation (WHO). This initial project identified the PSE policy documents for each organisation, with support from the United Nations Interagency Task Force (UNIATF) on the Prevention and Control of NCDs.

### Sample

We analysed the PSE policy documents from the ten major international organisations and development agencies identified by the WHO Global NCD Platform and CSIS in their original mapping exercise. The organisations were originally included based on their potential impact on the NCD and mental health agenda, assessed in terms of their international footprint, annual budget, and perceived intersection with NCD and mental health outcomes. All of the included organisations operate in at least 50 countries and have an annual operating budget of at least US$1 billion. The original inclusion and exclusion decisions were reached by consensus between the WHO Global NCD Platform and CSIS project staff, with additional input from UNIATF staff.

### PSE policy documents

Our research team started with a list of all publicly available PSE policy documents for each agency identified by UNIATF and designated focal points from each of the organisations complemented with additional web search using Goole. Our search used each organisation’s name (and commonly used abbreviations) with ‘private sector engagement’ and synonyms for the same e.g. ‘corporate engagement’, ‘business engagement, and ‘partnership policy’.

We included ‘internal’ policies that governed direct engagement between the organisation and private sector entities, as well as any ‘external’ policies that the organisations had developed for their Member States/partners. These documents had different names, but all focused on providing organisational guidance for how to engage with private sector entities. All included documents were being used to govern organisational PSE policy at the time of analysis, in April 2024.

### Analysis

We used a Word-based template to extract data in the form of direct quotes on the relevant policy/policies for each organisation, as well as the stated purpose, types, and processes of private sector engagement. We presented these organisational approaches narratively, supported by a summary table. To provide additional context, we summarised how each organisation’s work intersects with NCDs, drawing on previously published UNIATF summaries [15]. We presented our findings in two sections. The first focused on types of engagement, and the second situating these types of engagement within the broader context of each organisation’s PSE policy.

To identify cross-cutting themes and assess the degree of alignment across the policy documents we used thematic analysis, following the approach described by Braun and Clarke [16]. We read and re-read the policy documents to become familiar with them. We used NVivo to generate initial codes. We adopted an iterative approach to combining codes into themes, moving back and forth between coding and analysis. We reviewed our themes and used thematic triangulation to assess the degree of alignment across the ten organisations’ PSE approaches, reporting areas of agreement, dissonance, and silence [17, 18].

## Results

### Types of engagement

Table 1 summarises the PSE documents for each of the ten organisations, the types of engagement that each document sets out, and a brief summary of the organisational relevance to NCDs. Looking across the documents, we found three broad categories of engagement types; partnerships; private sector contributions; and influencing activities.

**Table 1:** Private sector engagement

Policies and purpose	Types of engagement	**Organisational relevance to NCDs**
**FAO** Strategy for Private Sector Engagement 2021–2025 [19] ***Purpose of engagement*** Improve understanding of development issues to design better solutions; promote economic and social inclusion; foster innovation; facilitate inclusion to more profitable markets; develop the skills and capacities of smallholder farmers and other small and medium-sized enterprises (SMEs); support the private sector to recognise its social responsibility to contribute to the food and nutritional security of the population; and promote sustainable investment	***Policy dialogue***: engagement modality in which the private sector takes part in multi-stakeholder policy dialogues on complex development challenges in the areas of agriculture, the environment, natural resources, food security, and nutrition, under FAO’s mandate ***Capacity development***: the private sector contributes to FAO’s numerous capacity development activities, including those targeted at farmers, producer organisations, cooperatives, and SMEs, towards enhancing agricultural value chains ***Resource mobilisation***: a type of engagement in which the private sector makes financial or in-kind contributions to support FAO’s programs and projects ***Technical cooperation***: engagement mode aimed at leveraging private sector partners’ expertise, technical know-how, and experiences to design and deliver solutions to specific challenges ***Knowledge and research***: the private sector contributes to FAO’s knowledge and research capacity, including through the provision of data and information on market trends and emerging technologies ***Advocacy and communication***: collaboration between FAO and the private sector (e.g., the media industry) aimed at disseminating information and best practices related to key FAO priorities ***Innovation***: to leverage the private sector’s innovation capacity towards ensuring that FAO applies modern science and technology in response to new situations and challenges ***Data sharing and dissemination***: type of engagement consisting in the sharing and dissemination of private sector data and information through global networks ***Support for financing and investment***: to leverage private investment in food and agricultural systems and rural development, towards the achievement of the SDGs	FAO’s mission of defeating hunger and ensuring that people have access to enough high-quality food to lead active, healthy lives is directly aligned with preventing NCDs FAO is uniquely positioned to contribute to global efforts to reduce the prevalence of overweight, obesity and NCDs through the support it provides to countries in reforming their food systems and its work with line ministries responsible for agriculture, trade, environment, and rural development, as well as with other UN agencies and development partners FAO's capacity development efforts can help strengthen the resilience and livelihoods of people living with or affected by NCDs through skills, knowledge, tools, and resources to cope with the challenges posed by their health conditions
**Gavi** *Private Sector Engagement* Approach [20] ***Purpose of engagement*** Leverage the private sector’s expertise, resources, and innovation capacity to increase immunization impact while improving efficiency and sustainability, through activities such as resource mobilization, capacity development, and advocacy	***Financial contribution***: A financial pledge from a private sector actor to support Gavi’s programmes that may be matched by the Gavi Matching Fund – a public–private funding mechanism that doubles private sector partners’ investments in immunization ***Leveraged investment partnerships:*** A financial pledge accompanied by industry expertise/know-how or programmatic intervention (e.g. awareness raising), with a potential match by the Matching Fund ***Shared value partnership***: A long-term ‘win–win’ operational partnership where the private actor is fully integrated within Gavi’s model (e.g., vaccine availability, cold-chain equipment, supply chain services, capacity strengthening)	Gavi’s work on health system strengthening is vital for providing the structures that are needed to deliver high-quality NCD care, especially through the strengthening of primary care systems Gavi has called for a new approach to women’s healthcare to address NCDs and launched a taskforce on women’s health that advocates for a gender-based, lifecycle approach to NCD prevention and treatment Provides funding and investments to support the development and implementation of NCDs related programs such as vaccination against diseases that can lead to NCDs (e.g. HPV vaccination to prevent cancer)
**Global Fund** Framework on Private Sector Engagement [21] ***Purpose of engagement*** Create shared value in alignment with the Organisation’s mandate to reduce the health and economic burdens of AIDS, TB, and malaria, by mobilizing the private sector’s expertise and resources for supply chain enhancement, capacity building, improved performance, and advocacy	***Financial contributions***: these comprise both funds towards overall replenishment, as well as targeted funds towards a specific board-approved grant ***Non-financial or in-kind partnerships***: private sector actors provide non-financial support, including, among others: pro bono expertise and services for capacity building initiatives and performance improvements; advocacy support (e.g., awareness raising and behavioural change programs); ***In-kind donations of non-health products:*** subject to compliance with internal requirements that ensure that Global Fund’s objectives and interests are not compromised	HIV, tuberculosis and malaria are all chronic conditions that require ongoing clinical management and long-term supplies of medications Investing in primary care services and wider health system infrastructure required to manage (often comorbid) NCDs
**SDC** Handbook on Private Sector Engagement [22] ***Purpose of engagement*** Leverage the private sector's expertise and resources towards the achievement of the SDGs, by enhancing innovation settings and achieving greater impact, developing new approaches and instruments suitable for complex and fragile settings	***Development project-oriented PSE formats:*** engagement modalities that follow a traditional development project logic. For example, an initiative co-funded by the SDC and one or multiple private sector partners (and possibly other stakeholders) ***Financial market-oriented PSE formats:*** engagement modalities that follow an investment logic with high potential. These include two sub-categories: Grant-based instruments, which are non-refundable contributions aimed at facilitating private investment with development objectives (e.g., technical assistance facilities, impact bonds, social impact incentives); Return-based instruments, for which repayments are envisaged or at least possible (e.g., shares, loans, stakes in structured funds and guarantees)	SDC’s poverty-alleviation work includes a number of projects aimed at tackling NCDs – a major cause of inequality and impoverishment Projects include health promotion and disease prevention programmes, early detection programmes, investment in access to medicine and health technologies, and a range of mental health programmes SDC work to strengthen health systems in partner countries, increasing access to medicine and health technologies and focusing on health promotion and NCDs as a cost-effective approach
**UNDP** Policy on Due Diligence and Partnerships with the Private Sector [23] ***Purpose of engagement*** Harness the private sector’s core competencies, skills, and knowledge towards the achievement of UNDP’s targets, including the ones related to sustainable and inclusive economic growth through the creation of jobs for the poor; promotion of women’s empowerment; reduction of the likelihood of conflicts and natural disasters; and recovery in post-conflict and post-disaster situations	***Advocacy and policy dialogue***: engagement modality aimed at influencing business practices and encouraging the private sector to adopt more responsible and sustainable approaches ***Resource mobilization***: to leverage the private sector’s financial and in-kind resources to support UNDP portfolios, programs and projects ***Innovations***: type of PSE aimed at developing and implementing innovative solutions to address development challenges towards the achievement of the SDGs ***Core business for inclusive market development***: to leverage the private sector’s “core business strengths” (e.g., expertise, know-how, services, technology) for the implementation of inclusive market models ***Transformational partnerships***: these consist in complex multi-stakeholder and multidimensional partnerships to address systemic issues on a broad scale Other types (Responsible party and crowdfunding)	UNDP focuses on strengthening national capacity, leadership, governance, multisectoral action and partnerships to accelerate country NCD responses Access to basic services, poverty and inequality reduction, environment and energy, good governance, sustainable financing, gender, and south-south and triangular cooperation all intersect with NCDs Additional work focuses on the links between NCDs and poverty
**UNHCR** Private sector fundraising and partnerships [24] Evaluation of UNHCR’s Engagement with the Private Sector [25] ***Purpose of engagement*** Provide goods and services to refugees and improve their living environment; promote economic and financial inclusion of refugees (e.g., access to work permits and bank services); improve UNHCR’s operational efficiency; and encourage more refugee-inclusive business models and practices	***Fundraise and receive***: type of engagement where UNHCR solicits and receives financial or in-kind donations (goods or services) from private sector actors ***Exchange***: private sector partners address a challenge or need identified by UNHCR in exchange for benefits to their core business (e.g., a banking company offers financial accounts or loans to refugees) ***Integrate***: engagement modality in which UNHCR and private sector partners combine expertise and resources for joint value creation and mutual benefits (e.g., developing a platform to improve refugee employability) ***Transform***: UNHCR aims to influence and transform private partners'practices (e.g., encourage cessation of investment in fossil fuels). Changes in business practices may also lead to broader social change	UNHCR directly provides NCD services to millions of refugees and displaced people who do not have access to any other services UNHCR facilitates the integration of refugee NCD programmes into national systems with a focus on improving quality, accessibility and affordability; ensuring the rational use of medicines; and strengthening the clinical and community-based management of NCDs
**UNICEF** *Private sector partnerships: Engaging with businesses for impact* [26] ***Purpose of engagement*** Leverage data, expertise, assets, technology, communications, and outreach to improve the lives of children	UNICEF sets out a ‘wheel of engagement’ to divide engagement activities into four domains: ***Financing*** through strategic grants, in-kind contributions (products), and engaging employees & customers in fundraising activities ***Business & Practices*** through market shaping, influencing child rights in business, and encouraging reforms to company policies & guidance ***Advocacy*** through raising awareness, influencing the policy sector and governments, and agenda setting through platforms—in each case raising awareness of UNICEF priorities ***Core business & assets*** through co-creation around programme delivery, soliciting private sector expertise, procurement of products and services, and innovation in terms of delivering new products & services	Programmatic focus on NCD prevention and control for children and youth Improving immunization, particularly against human papillomavirus (HPV) Preventing low birth weight, stunting, and other forms of malnutrition, including overweight and obesity Investing in early childhood development Addressing adolescent physical health, mental health, and nutrition
**USAID** Private-Sector Engagement Policy [27] ***Purpose of engagement*** Leverage the private sector's expertise, resources, and investment in addressing health and development challenges; seek market-based solutions for greater sustainability; expand opportunities for American firms; and make more catalytic use of USAID’s resources	***Information sharing and strategic alignment***: engagement mode that aims to identify common interests and share knowledge and experiences ***Advancing learning and market research***: to conduct shared market research, joint strategic planning and project design, and enhance good practices for private sector engagement ***Harnessing private sector expertise and innovation***: to leverage industry expertise, research and innovation capacity, technology, and entrepreneurial skills, towards achieving development outcomes ***Catalyzing private sector resources***: type of engagement that mobilizes private sector resources—both financial and non-financial—to address a specific issue ***Unlocking private investment***: to encourage private sector investment in new models and solutions to development challenges, particularly interventions that can be replicated or brought to scale ***Strengthening the enabling environment***: engagement around issues of importance to US businesses, including policies and regulations, compliance with standards, and government capacity building	USAID’s approach to NCDs emphasises investments in health systems strengthening Integrating activities targeting NCDs into other global health efforts, particularly through technical assistance and research Developing national policies, strategies, and implementation tools in NCD prevention and control Collaborations with the World Diabetes Foundation to support the Ethiopia Diabetes Association to strengthen diabetes care and prevention Support to mental health programs, focusing on community-based approaches and integrating mental health services into existing health systems
**World Bank** Procurement Regulations for IPF Borrowers [28] Procurement guidance: How to identify and manage conflicts of interest in Evaluation Committees [29] ***Purpose of engagement*** Partnering with the private sector to unlock investment and leverage expertise to address complex global problems, end extreme poverty, and build shared prosperity	***Creating markets and matching opportunities****:* identifying new opportunities and unmet demand, sharing this information with the global business community, and helping governments attract investment ***Building new markets for companies****:* linking business interests with development impact in high-risk, fragile, and conflict-affected environments. Helping businesses navigate challenging situations by connecting them with local communities and supply chains. Providing political risk insurance, credit enhancement instruments, and guarantees **Promoting investment opportunities for investors and financial institutions**: mobilizing resources from capital markets **Working as development partners: **The private sector partners with the World Bank in addressing development challenges through ESG initiatives. Companies and philanthropic organizations collaborate through dialogues, knowledge exchange, and implementing on-the-ground initiatives	Provision of financial support, policy advice and technical assistance to strengthen the health system response and address key risk factors for NCDs Analytic work to understand the nature and magnitude of the NCD problem, identify risk factors and propose mitigation measures Expenditure analyses to determine the efficiency of public spending and identify strategies to expand domestic resource mobilization (including through taxation of health-harming products) to increase those available for primary health care services
**WHO** Framework of Engagement with Non-State Actors [30] Handbook for non-state actors on engagement with the World Health Organization [31] ***Purpose of engagement*** Accelerate progress towards global health goals; encourage NSAs to use their own activities to protect and promote public health, by intervening on the social, economic, environmental, and commercial determinants of health; and enhance compliance with WHO’s policies, norms, and standards	***Participation***: engagement modality in which non-state actors take part in various types of meetings organized by WHO. The nature and format of their participation depends on the type of meeting concerned (meetings of the governing bodies, consultations, hearings, and other meetings such as information sessions, briefings, scientific conferences, and multi-stakeholder platforms) ***Resources***: type of engagement in which private sector actors make financial or in-kind contributions to support WHO’s programs and projects. In-kind contributions include donations of medicines and other goods and free provision of services ***Evidence***: NSAs share their up-to-date information, technical know-how, and relevant experiences with WHO, by contributing to the Organisation’s research and evidence generation activities ***Advocacy***: collaboration between WHO and the private sector aimed at increasing awareness of health issues to encourage behavioural change and promote collaboration between NSAs where joint action is required ***Technical collaboration***: engagement modality in which NSAs provide technical support to any activities falling within WHO’s General Programme of Work, including: (1) product development; (2) capacity building; (3) operational collaboration in emergencies; and (4) contributing to the implementation of WHO’s policies	WHO provides global strategic leadership on efforts to tackle NCDs Regional and country-level support in identifying priorities and tailored solutions Identifies highly cost-effective ‘Best Buy’ interventions for NCDs Numerous country programmes to address a wide range of NCD issues, including direct support to health ministries

Partnerships: Three main subthemes emerged in this domain. Two of the PSE strategies talked about **two-way data sharing** and wider dissemination (FAO and USAID) to support the objectives of the international organisation. Four of the documents discussed **joint advocacy and dialogue** on shared issues (FAO, UNDP, UNICEF, WHO). For instance, WHO deliberately seeks to engage a wide range of non-state actors in global policy meetings. Five of the organisations talked about **integrated delivery of services** (UNHCR, SDC, WHO, FAO, UNDP). UNHCR, SDC, and WHO use language around technical cooperation to jointly fund and deliver health and development programmes. FAO and UNDP refer to deeper **‘shared value’ and ‘transformational’ partnerships**, respectively, reflecting long-term, multidimensional collaborations that seek to address systemic issues on a broad scale.

Private sector contributions: Six subthemes emerged in this domain. The first is straightforward **financial contributions**, where a private sector entity makes a monetary donation or investment. Interestingly, only the Gavi and the Global Fund policies identified this as a standalone engagement type. The FAO, Global Fund, UNDP, and USAID all mentioned **in-kind contributions**, spanning the provision of products, technology, expertise, and technical cooperation. UNDP, UNHCR, UNICEF, and WHO referenced a third type of **bundled contribution**, where financial and in-kind resources are donated to the international organisation. FAO and USAID documents also talk about using PSE to **leverage private support from other sources**, i.e. working together to raise additional investment from other private sector actors. FAO and Gavi discuss **capacity development as well**—engaging with private sector actors to develop skills, markets, and systems that support areas like farmers’ abilities to grow and sustain productive crops. The final engagement type, mentioned by FAO, UNDP, USAID, and WHO, was around **research and innovation**, drawing on private partners’ resources, skills and expertise to generate new knowledge and improve existing projects and systems.

Influencing activities: The final meta-theme was around engagement activities designed to influence or support businesses. The first subtheme emerged from the UNHCR and UNICEF policies that include deliberate strategies to proactively **redress harmful business practices** and ‘advocate’ for better policies and regulations in sectors where industries are currently harming health and human development, such as fossil fuel businesses. The other subtheme was around **creating market opportunities** for firms seeking to operate in challenging (e.g. conflict-afflicted) environments, through de-risking investment and supporting the development of business-friendly policies and practices. This theme was only mentioned by USAID and the World Bank.Organisational approaches.

**Gavi** engages with the private sector for supply chain enhancement and demand generation, aiming to increase vaccine coverage and equity by identifying new ways to reach populations that lack access [20]. As an example, since 2014 the UPS Foundation has been providing funding and technical expertise to establish reliable cold chains, enhance last-mile delivery of vaccines, and increase the number of trained supply chain managers in several priority countries [32]. The partnership was initiated by Gavi and resulted in UPS loaning one of its experts to advise on the organisation’s supply chain strategy [33]. An independent evaluation of Gavi’s overall approach concluded that while the organisation has managed PSE satisfactorily, key issues remained around project implementation and performance risks; political and reputational risks; cyber security risks; donor dependency and fund flow risks, among others [34].

***The Global Fund***’s PSE Framework serves as an instrument for identifying the risks and benefits through a risk management process with clearly identified roles and responsibilities for decision-making, based around a simple categorisation of PSE engagement types (financial and non-financial), excluding in-kind donation of health products [21]. The Fund has been involved in several high-profile private sector partnerships, including independent projects with Heineken and Coca-Cola to support the last-mile distribution of medicines and to generate demand. These partnerships mostly involved the provision of technical assistance and expertise from the two companies [35]. Working closely with an alcohol producer and a firm whose products are harmful to health has raised significant controversy [36]. The Global Fund suspended its relationship with Heineken over the company’s use of women to sell its products in a way that was considered sexually exploitative [37]. The experience resulted in the organisation updating its Framework to include additional safeguards and risk mitigation strategies. For example, the updated document outlines a risk-screening process that reviews business, environmental, human rights, and gender policies and practices, as well as political exposure, privacy, and data security. The Framework emphasises the need for robust due diligence, transparency and accountability. Cooperation with the tobacco and arms industries is prohibited. Additionally, the Global Fund exercises careful judgment when considering collaborations with private sector entities whose policies or actions may have detrimental impacts on human health, including considerations related to both communicable and NCDs, as well as their determinants [21].

***FAO*** lists a large number of potential engagement approaches in its PSE policy document [19]. The organisation has committed to offering a “proactive” outlook to due diligence, facilitating the formulation and implementation of partnerships, as opposed to a “defensive” approach that recognizes the need for decentralized decision-making on risk management [19]. Their strategy stipulates a set of risk categories for private sector engagement, as well as an accountability framework for its risk assessment process, and clear internal guidelines for its personnel. Partnerships are monitored to assess if the balance of risk versus benefit is maintained or whether measures to mitigate risks and/or terminate engagement are needed. FAO’s exclusionary criteria include businesses that fail to meet in practice the principles of the UN and are associated with armaments, weapons, tobacco, pornography, human rights violators, forced and child labour. FAO has recently launched a “Connect” portal to serve as an official channel for interacting with prospective and current partners, providing an online repository of partner information “based on transparency, openness, inclusivity, accountability, integrity, and mutual respect” [19]. Among the documents available on the platform are partnership agreements and memoranda of understanding, work plans, and letters of intent. In addition, FAO has established a dedicated Private Sector Advisory Group to provide advice on implementing the organisation’s Strategy for Private Sector Engagement [19]. As part of its approach, FAO aims to address a “cultural gap” leading to “an inherently conservative attitude toward private sector engagement,” while at the same time improving procedures for due diligence and risk mitigation [19]. FAO’s proactive and robust PSE approach is grounded in a position of aiming to engage with the broadest possible array of private entities through extremely transparent processes. Their stance includes engagement with manufacturers of highly processed foods, as long as the process and outcomes are clear, transparent, and accountable [38]. For instance, recently FAO signed a Memorandum of Understanding (MoU) with Mars (a multinational manufacturer of confectionery, pet food, and other food products) to promote better understanding and application of Codex Alimentarius international food safety standards. [39]

***UNHCR***’s PSE policy sets out four different modalities of engagement through which the organisation solicits and receives financial or in-kind donations from private sector actors [24]. UNHCR does not have specific risk management processes and a 2019 evaluation recommended establishing a ‘Partnering Support Service’ to provide technical assistance and training on PSE, and to ensure greater coordination and coherence within the organisation [25]. The evaluation also highlighted the need to improve staff negotiating skills and their ability to select appropriate partners and types of engagement, as well as the importance of establishing “formal and informal spaces for sharing and learning across teams and operations on how others engage with the private sector.” [25].

***The World Bank*** does not have a publicly available PSE policy. Their webpage ‘World Bank and Private Sector’ sets out four types of engagement aimed at “accelerating sustainable and inclusive economic growth across regions and sectors” [40]. The Bank strongly advocate for proactive engagement with the private sector, arguing that the scale and complexity of the world’s challenges require collaborative action [41]. It has set up the ‘Private Sector Investment Lab’ to help mobilise and direct private capital to address pressing global issues [42]. The Bank has issued an operations manual on performance standards for private sector activities which is used to guide decisions around financing private sector-led projects [43]. This document does not provide guidance on whether to proceed with a given project, or principles to guide due diligence and conflict of interest assessments.The Bank has also produced guidance to support borrowers in identifying and managing actual, potential, or perceived conflicts of interest during procurement processes. This guidance document proposes a three-step approach for managing financial conflicts of interest, revolving around obtaining formal declarations from all parties concerned, independent review of these self-declarations, and injunction to manage and resolve any identified conflicts in a way that is ‘appropriate, fair and transparent’ [29]. No other risks (e.g. reputational) are mentioned in the guidance [28]. The Bank has also produced reports for emerging markets and multilateral development banks that offer guidance on how to engage with the private sector, however, these do not represent corporate policy documents [44, 45].

***WHO*’s** PSE approach is governed by its *Framework of Engagement with Non-State Actors*. [30] Engagement can include participation in WHO meetings, financial or in-kind contributions, or exchange of evidence, information and technical innovations, as well as know-how, when appropriate. WHO does not engage with the tobacco and arms industries or entities with ties to tobacco and arms. Due diligence is conducted before engagement with any non-State actor, including commercial entities. WHO has initiated dialogues with a variety of private sector entities to advance the NCD agenda, including economic operators in alcohol production and trade, the food and non-alcoholic beverage industry, the pharmaceutical industry, and the leisure and sports industry [46, 47]. While the Organisation conducts dialogue with the alcohol industry, it does not engage in any formal collaboration [30, 48]. In its dialogues, the organisation sets specific expectations for each industry. For example, calling the food and beverage industry to adopt standardized sodium targets for specific food categories and reformulate products accordingly [46]. The organisation also called on the sports industry to contribute to the effective implementation of the Global Action Plan on Physical Activity 2018–2030 by promoting physical activity in the workplace, supporting improved physical education for children, and extending services outside the formal gym and club environments [49, 50]. In addition, the WHO periodically convenes expert groups at the global level to advance private commercial sector commitments towards creating healthier environments. For instance, in May 2021, the Organisation released global sodium benchmarks for a variety of foods [51]. WHO has recently launched a decision-making toolkit for Member States on how to engage with the private sector to scale up action for NCD prevention and control in countries [52]. WHO has also established a ‘Global Noncommunicable Diseases Platform’ uniting the Global Coordination Mechanism on NCDs and the United Nations Interagency Task Force on NCD prevention and control to facilitate multisectoral and multistakeholder engagement.

***UNDP*** ‘s *Policy on Due Diligence and Partnerships with the Private Sector* governs how the organisation relates to commercial entities [23]. Its risk assessment tool for PSE recommends starting with the identification of the benefits and risks to determine the value of the engagement, defining an appropriate level of “risk tolerance,” and conducting due diligence to identify any other potential conflicts or areas of concern [53]. The policy document aligns with the organisation’s *2023–2025 private sector development and partnership strategy* [54]. The agency recommends researching potential private sector partners both through publicly available sources and by collecting information directly from companies via meetings and questionnaires. UNDP’s policy highlights that due diligence should be even more rigorous when considering engagement with high-risk industry sectors that may have negative impacts on people or the environment, such as the alcohol industry, extractive, power generation, and construction industries, as well as fast-food chains and sugar-sweetened beverage manufacturers [53]. Further, the agency notes that different levels of due diligence are required depending on the type of engagement. For example, advocacy engagement with low-risk industry sectors requires less rigorous due diligence than engagement around core business operations and value chains, for which comprehensive reviews for both low- and high-risk industry sectors are necessary [53]. UNDP provides guidance on whether or not to engage in cases where subsidiaries, parent companies and distributors or suppliers (including those of a corporate foundation’s ‘founding’ or ‘host’ company) are engaged in activities that fall under exclusionary criteria. However, no policy or recommendations were found for how Member States should engage with the private sector. In terms of NCD implications, UNDP's approach is relatively conservative and applies strict criteria for engagement with ‘high-risk’ sectors such as alcohol, fast foods, and sugar-sweetened beverages. Engagement with the tobacco industry is prohibited.

**UNICEF**’s 2022–2025 Strategic Plan is focused around ensuring that every child survives and thrives, highlighting NCDs as one of the core areas of work [55]. UNICEF has several collaborations with private companies like BEKO, MSC Cruises, and Novo Nordisk focused on NCDs (i.e. fighting childhood obesity, addressing malnutrition, optimising youth mental health, and strengthening health systems) [56]. The organisation’s PSE strategy identifies partnership and engagement with private sector actors (among others) as an important change strategy to accelerate progress and sets out the ‘Business for Results’ agenda that aims to systematically integrate businesses as a programming strategy [55, 57]. Building and maintaining partnerships is one of eight core competencies expected of all UNICEF staff [58]. The agency states that engagement can help to leverage data, expertise, assets, technology, communications and outreach to improve the lives of children [55, 59]. In the joint UNICEF-UNIATF document ‘Responding to the challenge of NCDs’, UNICEF states that due diligence is required “to ensure that desired results are obtained” when engaging with private sector entities who market tobacco, alcohol, unhealthy foods and beverages [59]. They note that some pharmaceutical partnerships may also pose potential or real conflicts of interest [59]. The agency has developed internal policy documents that provide an approach, criteria, and procedures for engaging external partners [26, 60] and an internal-facing online course that unpacks UNICEF’s approach to PSE in further detail [61]. Their approach is summarized by the social and behavioural change unit in a publicly available document on how to engage with businesses for impact [26]. The document outlines three core implementation steps, including identifying the link between business and the problem, exploring which businesses can support progress toward the objectives, and determining how business engagement can be effectively integrated with reference to four domains in the “wheel of engagement.” These domains encompass financing, advocacy, business practices, and core business and assets. No document was found from UNICEF provideings PSE guidance to Member States.

**SDC** has published a PSE Handbook to guide engagement when there is co-ownership and co-funding of a given development intervention. It documents two main types of engagement modality: development project-oriented and financial market-oriented. SCD also set out a *Partner Risk Assessment* approach, with more guidance provided in the specific ‘PSE Risk Management Process’. To improve the effectiveness of private sector engagement, the agency has also established the Competence Center for Engagement with the Private Sector in 2017 [22]. Lessons learned from the agency’s experience include the need to pay special attention to bridging different cultures and mindsets of partners. For example, public sector entities may be concerned about reputational risks leading them to institute overly-complex administrative procedures, which can become cumbersome for private sector actors. Thus, the agency recommends that all parties involved ‘step out of their comfort zone’, adopt internal policies and procedures, and be willing to undertake risks, while at the same time adopting appropriate safeguards and risk mitigation strategies [22]. The agency, provides guidelines how to asses a PSE prospect and screens them against an exclusion list. SDC does not partner with PSEs involved in excluded sectors or practices. For instance, PSEs are excluded if they directly engage in the production or trade of weapons, certain alcoholic beverages, tobacco, or gambling. They’re also excluded if they participate in illegal activities, harm UNESCO World Heritage Sites, deal with certain hazardous substances, or use certain harmful practices in forestry and fishing. Sustainability commitment and Environmental, Social, and Governance risk are also additional points for discussion that SDC considers.

**USAID**’s PSE policy seeks to leverage private sector resources and encourage private sector investment in new models and solutions to development challenges. In recognition of the role of the private sector in delivering development and humanitarian programmes—including those relating to NCDs—the USAID PSE policy represents an agency-wide mandate to enhance its work with the private sector, signalling an intentional shift to pursue market-based approaches and investment to support countries development and progress on the ‘journey to self-reliance’. This shift to an enterprise-driven development model is intended to enable USAID to play a more catalytic role in achieving results, as opposed to taking on the responsibility of designing, funding, and managing its projects. USAID’s risk management process relies on the view that the “potential benefits of PSE outweigh the potential costs and the possibility of failure.” According to the agency, comprehensive due diligence conducted early on in the engagement can effectively mitigate reputational and fiduciary risks for international organisations [27]. Risks are identified by considering the relevant systems, markets, and relationships, and then weighed against the probability that risk could occur. The policy also sets out good practices for mitigating risks in PSE and supports identifying and filling gaps in knowledge, and research and evaluation through the ‘PSE Evidence and Learning Strategy’. In its Private-Sector Engagement Policy, USAID highlights the importance of assessing past performance, reputation, policies, and future directions of potential private sector partners [27]. When researching a given company, USAID recommends considering the following aspects: added benefits of the engagement, shared ethics, the company’s responsible business practices, environmental protection policies, as well as respect for human rights. No document was found that provides guidance to USAID’s partners on how they should engage with private sector entities.

## Discussion

In this study we reviewed the policy documents that govern private sector engagement at ten international organisations whose work intersects with NCDs. All ten agencies have different agendas, strategic priorities, and purposes for engaging with the private sector which manifests in different approaches, frameworks, and modalities. At a basic level, there is broad conceptual alignment in the recognition that robust risk assessment and due diligence processes are required to prevent financial conflicts of interest. However, once the level of risk has been identified, the organisations vary in their appetite for risk and approach to engagement, mainly based on culture, past experience, and aims. The framing, processes, and content of their policies varies widely.

There was broad alignment across the ten organisations’ PSE policy documents in terms of acknowledging the need to engage with the private sector in order to deliver on their individual mandates and achieve the wider SDGs. Virtually all organisations use some form of due diligence and risk stratification to guide their engagement activities and distinguish between financial, in-kind, and other forms of engagement. Only a subset of the policies identify prohibited industries. In terms of dissonance, whilst the policies all acknowledge the need for due diligence, there was marked variation in the framing, processes, and detail provided. USAID and the World Bank’s PSE approaches stood out as much more proactive and pro-business. Both organisations aim to facilitate investment and productivity growth as a means and end in itself, and view private sector engagement as ‘catalytic’, with an eyes-open appetite for risk. The Bank does not have any publicly available internal PSE policy, and the USAID document focuses exclusively on financial conflicts of interest, whereas other organisations consider reputational and other types of risk (performance, cyber, political, donor dependence etc.).

At the other end of the spectrum are organisations with a more conservative approach to PSE, including FAO, WHO, UNDP, and the Global Fund. These organisations stress the need for comprehensive risk assessment, and in the case of WHO, UNDP and FAO proscribe any kind of engagement with a range of industries such as tobacco, arms, and alcohol. In the middle of the spectrum are GAVI, UNHCR, UNICEF and SDC who proactively engage with damaging sectors in order to try and mitigate the harm they cause, based on thorough due diligence procedures. The sophistication and level of detail varies markedly across the policy documents, with organisations like the SDC providing very detailed guidance on all aspects of managing private sector partnerships. Whilst many of the more detailed policies cover similar themes, the frameworks, terminology, and content was unique in every instance. Whilst each organisation had a policy that governed how the organisation should structure direct private sector engagement, only the WHO and the World Bank have produced guidance for their Member States [45, 52]. WHO has developed a tool for Member States on how to engage with the private sector for broader NCD and mental health agendas. No other organisations had specific guidance for engaging with private entities around NCD activities, although WHO, FAO, UNDP and the SDC do single out particular sectors that carry the highest reputational risks e.g. tobacco and arms.

In terms of risk management, all of the policies acknowledge that different entities pose differing levels of risk, be they reputational, fiduciary, legal, or market-based. Risk appetite varies widely, with USAID occupying the least conservative end of the spectrum, partially as a result of its broad mandate to address health and development issues using market-based solutions and expand opportunities for American firms. The UNDP PSE policy specifically outlines four further broad risk categories: misalignment of core values and objectives; reputational risks by association with private sector entities with unethical practices; damage to perceived impartiality by being seen as endorsing a particular business, product, or service; market distortions caused by giving an unfair advantage to a particular entity; and negative publicity because of a private sector partner’s action [53].

Three of the organisations (the Global Fund, FAO, UNDP and WHO) have effectively identified a set of sectors that they will not engage with. UNDP further defines ‘high-risk’ industries that require a different tier of due diligence. It is perhaps not surprising that organisations with mandates that focus exclusively on health are more risk-averse. Despite this heterogeneity, all of the included organisations recognise that robust and transparent mechanisms are required to reduce risk and maximise impact—and that these processes impose their costs on participants. The SDC has explicitly sought to streamline due diligence processes, recognising the cultural disconnect between development and private sector partners. FAO has also taken proactive steps to maximise opportunities for engagement with a broad array of private sector partners but within the context of total transparency and clear processes. UNHCR has identified issues with robust but onerous central due diligence processes that can impede productive engagements at the local level. There is a ubiquitous tension between devolving PSE decision-making and minimising organisational risk exposure. FAO, the Global Fund, SDC and USAID stress the importance of ongoing processes to monitor the changing nature of the risks associated with partnerships.

Appropriate risk assessment and mitigation strategies are essential for international organisations to leverage the expertise and resources of the private sector while protecting themselves from any undue influence and conflicts of interest [62]. When the essential safeguards are put in place, and appropriate governance structures, regulatory frameworks, and accountability and transparency mechanisms are implemented, PSE can contribute to the NCD agenda [63], particularly in LMIC contexts where the needs are great but public resources are limited [64, 65].

There is a ubiquitous tension between devolving PSE decision-making and minimising organisational risk exposure. FAO, the Global Fund, SDC and USAID stress the importance of ongoing processes to monitor the changing nature of the risks associated with partnerships. In addition to risk assessment and due diligence, all reviewed international organisations and development agencies emphasise the importance of developing solid accountability and transparency mechanisms to ensure that all parties live up to their commitments. Enhancing accountability and transparency requires establishing flexible multi-stakeholder platforms to ensure equitable representation of all parties involved.

The alignment of PSE policies across international organisations is not only a technical matter, but also a strategic imperative for advancing the SDGs. As noted by Dumitriu, policy coherence in UN-private sector partnerships is essential for realising sustainable development at scale [12]. International organisations are increasingly looked to as normative leaders, with their policies shaping national approaches to public–private collaboration [66]. Furthermore, the growing importance of blended finance and catalytic capital has made clarity around risk governance and engagement modalities a pressing issue for donors and partner governments alike. [67]

There is emerging evidence that well-aligned and clearly communicated PSE policies can enhance trust, improve collaboration, and reduce transaction costs across multi-stakeholder partnerships [68]. However, in the absence of shared standards, fragmentation in PSE approaches may undermine collective efforts to tackle complex global health challenges, including NCDs [64, 69, 70]. This study provides an empirical basis for identifying areas of alignment and divergence in current PSE frameworks, offering a foundation for future harmonisation efforts across the global development system.

To foster greater coordination and coherence, three strategic actions may be particularly impactful. First, the development of a shared *reference framework*—akin to the UN Joint Inspection Unit's proposal for principle-based partnership standards [12]—could provide common terminology, engagement typologies, and minimum safeguards. This would not override agency mandates but offer a scaffold for alignment. Second, enhanced inter-agency learning platforms (e.g. via the UNIATF or the WHO Global Coordination Mechanism on NCDs) could facilitate systematic exchange of practice, joint training, and peer review of engagement strategies. Third, there may be value in establishing a light-touch monitoring mechanism, such as an annual voluntary self-assessment or scorecard to benchmark progress and encourage convergence without creating binding obligations. In a global landscape increasingly defined by geopolitical volatility, resource constraints, and contested multilateralism, such mechanisms could support the legitimacy and sustainability of PSE as a core tool for achieving health and development goals. Further research should seek to explore the impact of these policies on the way that international organsations engage with the private sector, with an emphasis on identifying policies and practices that maximise synergistic health outcomes whilst minimising risks.

This study has some limitations. Whilst the included organisations have a very large collective footprint in global NCD prevention and control, the underlying purposive sampling criteria were essentially subjective. As such, our central findings that all ten organisations use similar processes but gear their risk calculations differently is important but not necessarily generalisable. Our focus on publicly available documents means that we may have missed strategically important internal policies. Whilst our focus was on NCD-related PSE, none of the included policy documents specifically related to NCDs.

## Conclusions

By virtue of their overlap with other conditions and huge health, economic and social costs, NCDs have become a strategic priority for virtually all international organisations working in the field of health and development. As emphasised during the United Nations High-Level Meetings on the Prevention and Control of NCDs, private sector engagement is a core and unavoidable part of tackling the growing health and economic burden of NCDs. Leading international health and development agencies are producing organisational policies to optimise their PSE activities and whilst there is broad concordance in the acknowledgement of the need for due diligence processes, we found poor alignment in terms of the content, processes, sophistication, and overall framing of these policies. Alignment of approaches would potentially improve coordination and efficiency as development partners seek to harness the power of the private sector to advance the SDGs. The UNIATF Secretariat based in WHO’s Global NCD Platform has an important role to play in supporting future efforts in this domain.

## Data Availability

All data generated or analysed during this study are included in this published article.

## Abbreviations

CSIS: Centre for Strategic and International Studies
FAO: Food and Agriculture Organisation
GAVI: Gavi the vaccine alliance
NCDs: Noncommunicable diseases
PSE: Private sector engagement
SDC: Swiss development agency
SDG: Sustainable development goals
UN: United Nations
UNDP: UN development programme
UNHCR: UN Refugee Agency
UNIATF: UN interagency task force on the prevention and control of NCDs
UNICEF: UN Children’s Fund
USAID: US Agency for International Development
WHO: World Health Organisation
